# TIGIT^+^Tfh show poor B-helper function and negatively correlate with SARS-CoV-2 antibody titre

**DOI:** 10.3389/fimmu.2024.1395684

**Published:** 2024-05-29

**Authors:** Natalie M. Edner, Luke P. Houghton, Elisavet Ntavli, Chloe Rees-Spear, Lina Petersone, Chunjing Wang, Astrid Fabri, Yassin Elfaki, Andrea Rueda Gonzalez, Rachel Brown, Kai Kisand, Pärt Peterson, Laura E. McCoy, Lucy S. K. Walker

**Affiliations:** ^1^ Division of Infection and Immunity, Institute of Immunity and Transplantation, University College London, London, United Kingdom; ^2^ Queen Square Institute of Neurology, University College London, London, United Kingdom; ^3^ Institute of Biomedicine and Translational Medicine, University of Tartu, Tartu, Estonia

**Keywords:** follicular helper T cells, antibody response, TIGIT, B cell help, COVID-19

## Abstract

Circulating follicular helper T cells (cTfh) can show phenotypic alterations in disease settings, including in the context of tissue-damaging autoimmune or anti-viral responses. Using severe COVID-19 as a paradigm of immune dysregulation, we have explored how cTfh phenotype relates to the titre and quality of antibody responses. Severe disease was associated with higher titres of neutralising S1 IgG and evidence of increased T cell activation. ICOS, CD38 and HLA-DR expressing cTfh correlated with serum S1 IgG titres and neutralising strength, and interestingly expression of TIGIT by cTfh showed a negative correlation. TIGIT^+^cTfh expressed increased IFNγ and decreased IL-17 compared to their TIGIT^-^cTfh counterparts, and showed reduced capacity to help B cells *in vitro*. Additionally, TIGIT^+^cTfh expressed lower levels of CD40L than TIGIT^-^cTfh, providing a potential explanation for their poor B-helper function. These data identify phenotypic changes in polyclonal cTfh that correlate with specific antibody responses and reveal TIGIT as a marker of cTfh with altered function.

## 1 Introduction

Follicular helper T cells (Tfh) are important mediators of humoral immune responses, providing help to B cells for the production of high-affinity antibodies during germinal centre (GC) reactions (1). While Tfh exert their function predominantly in the B cell follicles of secondary lymphoid organs, blood-borne counterparts of Tfh cells exist, termed circulating Tfh (cTfh) (2, 3). Analysis of cTfh is an area of growing interest, with suggestions that these cells could potentially serve as biosensors for advanced immunomonitoring or predictive modelling (4–8). In the setting of autoimmunity, where inappropriate immune responses cause tissue damage, the frequency of cTfh has been shown to be elevated and can correlate with pathogenic autoantibodies (9–11). Similarly, following infections or vaccination, cTfh expressing activation markers, such as ICOS and CD38, are transiently increased and correlate with antibody levels (12–15).

To further explore cTfh phenotype in a setting of immune dysregulation, we have used cryopreserved peripheral blood mononuclear cell (PBMC) samples from the first wave of the COVID-19 pandemic in which exposure-naïve individuals responded to the original Wuhan strain of SARS-CoV-2. While the majority of infections resulted in only mild disease symptoms, some infected individuals developed more severe disease, requiring hospital care and admission to intensive care units (ICUs), and we have focussed on the latter individuals here.

Both the innate and adaptive arm of the immune system are known to be critical for control of SARS-CoV-2 infection and a dysregulated immune response has been linked to severe disease outcomes. Infection with SARS-CoV-2 also induces neutralising antibodies and CD4 and CD8 T cells responses, which orchestrate viral control (16–19). It has been suggested that in elderly individuals these three arms of the adaptive immune response are imbalanced providing at least a partial explanation for the higher risk of developing severe COVID-19 seen in this age group (18, 20). Neutralising antibodies, which often target the receptor binding domain within the S1 subunit of the viral spike protein, have been shown to correlate with protection from reinfection and severe disease and are the target for successful vaccination strategies (21, 22). An increase in activated cTfh has also been documented following SARS-CoV-2 infection (23–26).

In an interesting twist, humoral immunity can also compromise responses to SARS-CoV-2 as illustrated by the discovery that neutralising antibodies to type I interferon (IFN) can be found in about 20% of patients with severe COVID-19 (27, 28). Here, pre-existing autoimmunity dysregulates the innate antiviral response, interfering with the precisely controlled production of type I IFN required to orchestrate immunity. Notably, a delay or impairment of the type I IFN response has been associated with increased viral loads and severe disease (29–33) and several SARS-CoV-2 proteins have been found to antagonise components of the IFN response (34, 35).

In this study, we have applied high-dimensional immunophenotyping of PBMC samples from a cohort of hospitalised COVID-19 patients, focusing in particular on the cTfh compartment and how it relates to the anti-SARS-CoV-2 specific antibody response and disease severity. We found evidence of increased activation of CD4 and CD8 T cells as well as higher S1 IgG antibody titres and stronger neutralising antibodies in severe disease. Additionally, we observed that TIGIT expression by cTfh was inversely correlated with S1 IgG antibody titres. This prompted additional investigation into the nature of TIGIT expressing cTfh. Our findings demonstrate that TIGIT^+^ cTfh are skewed towards a Tfh1 phenotype and have a lower capacity to provide B cell help *in vitro*, potentially attributable to their inferior expression of CD40L.

## 2 Materials and methods

### 2.1 Study participant details

Samples of individuals hospitalised at Royal Free Hospital, London, United Kingdom, with COVID-19 were cryopreserved and stored in the Royal Free London Biobank (NRES 16/WA/0298). Samples were collected from April to June 2020. SARS-CoV-2 infection was confirmed by PCR-based testing and disease severity was assessed according to supplemental oxygen requirements and ICU admission in the duration of hospitalisation (see patient information in **Supplementary Table 1**). For three individuals no positive PCR test result could be obtained at the time of blood collection, however a diagnosis of SARS-CoV-2 infection was assigned by the treating physicians on the basis of clinical symptoms. In total, PBMCs from 36 patients (16 no suppl. oxygen, 12 suppl. oxygen, 8 ICU) and serum samples from a subset of these patients (2 no suppl. oxygen, 5 suppl. oxygen, 8 ICU) were obtained. The protocol and consent document of this study were approved by appropriate independent ethics committees or institutional review boards. Peripheral blood of self-declared healthy individuals was collected at the UCL Institute of Immunity and Transplantation, London, United Kingdom.

### 2.2 Sample preparation

Cryopreserved samples were thawed in a 37°C water bath and vial contents transferred to a 50 mL Falcon tube containing 10 ml pre-warmed C10 media (RPMI+ (RPMI 1640 (Thermo Fisher) + 2 mM L-Glutamine (Thermo Fisher) + 100 U Penicillin-Streptomycin (Thermo Fisher) + 50 µM 2-Mercaptoethanol (Sigma)) + 10% foetal calf serum (FCS, PAN-Biotech)). After centrifugation at 400 x *g* for 7 minutes cell pellet was resuspended in 10 ml pre-warmed C10 media.

Peripheral blood mononuclear cells (PBMCs) were isolated from whole blood by density gradient centrifugation. In brief, whole blood was diluted in phosphate-buffered saline (PBS, Thermo Fisher) and layered over Histopaque 1077 (Merck) in LeucoSep 50 ml tubes (Greiner Bio-One). After centrifugation at 800 × *g* for 15 minutes with no brake, supernatant above barrier was poured into new 50 ml Falcon tube and centrifuged at 350 × *g* for 10 minutes to remove remaining histopaque. Supernatant was discarded and pellet resuspended in PBS + 2% FCS (P2). Platelets were removed by centrifugation at 250 × *g* for 10 minutes. The resulting pellet was resuspended in P2.

### 2.3 Anti-IFN autoantibody assay

The sequences encoding IFNα subtypes (IFNα1, IFNα2, IFNα8, IFNα21) or IFNλ subtypes IFNλ1–3 (IL-29, IL-28A, IL-28B) were cloned into pPK-CMV-F4 plasmid (PromoCell GmbH) where NanoLuc luciferase sequence (Promega) was inserted instead of firefly luciferase. HEK293 cells were transfected with the constructs, 72 hours later cell media containing the secreted fusion proteins was collected. Patient sera were diluted 1:10 using buffer A (50 mM Tris pH 7.5, 100 mM NaCl, 5 mM MgCl_2_, 1% Triton X-100). 25 µL serum dilution and 25 µL of protein G agarose bead suspension (Exalpha Biologicals) was incubated in a 96-well microfilter plate (Merck Millipore) at room temperature for 1 hour. The next 1-hour incubation step was carried out after the addition of either IFNα subtype mix or IFNλ subtype mix of fusion proteins corresponding to 1 x 10^6^ LUs (luminescent unit) for each. A vacuum system (Millipore) was used to wash away the unbound fusion proteins. Nano-Glo^®^ Luciferase Assay (Promega) and VICTOR X Multilabel Plate Reader (PerkinElmer Life Sciences) was used to quantify luminescence. The same three AAB negative control serum samples were run in duplicates with each 96-well plate. For each sample a fold change of luminescence relative to the mean of three negative control samples was calculated by dividing the mean luminescence value of the test sample with the mean of the negative control samples.

### 2.4 Neutralisation assay

HIV-1 particles pseudotyped with SARS-CoV-2 spike were produced in a T75 flask seeded the day before with 3 million HEK293T/17 cells in 10ml complete DMEM, supplemented with 10% FBS, 100IU/ml penicillin and 100μg/ml streptomycin. Cells were transfected using 60μg of PEI-Max (Polysciences) with a mix of three plasmids: 9.1μg HIV-1 luciferase reporter vector (36), 9.1μg HIV-1 p8.91 packaging construct and 1.4μg WT SARS-CoV-2 spike expression vector (36). Supernatants containing pseudotyped virions were harvested 48 h post-transfection, filtered through a 0.45-μm filter and stored at -80°C. Neutralisation assays were conducted by serial dilutions of sera in DMEM (10% FBS and 1% penicillin-streptomycin) and incubated with pseudotyped virus for 1 h at 37°C in 96-well plates. HeLa cells stably expressing ACE-2 (provided by J.E. Voss, Scripps Institute) were then added to the assay (10,000 cells per 100μl per well). After 48–72 h, luminescence was assessed as a proxy of infection by lysing cells with the Bright-Glo luciferase kit (Promega), using a Glomax plate reader (Promega). Measurements were performed in duplicate and used to calculate 50% inhibitory dilution values in GraphPad Prism software.

### 2.5 Semiquantitative ELISA

As described previously (37), nine columns of a half-well 96-well MaxiSorp plate were coated with purified SARS-CoV-2 spike S1 protein in PBS (3μg/ml per well in 25μl) and the remaining three columns were coated with 25μl goat anti-human F(ab)’2 diluted 1:1000 in PBS to generate the internal standard curve. After incubation at 4°C overnight, the ELISA plate was blocked for 1 h in assay buffer (PBS, 5% milk, 0.05% Tween 20). Sera was diluted in assay buffer at dilutions from 1:50 to 1:5000 and 25μl added to the ELISA plate. Serial dilutions of known concentrations of IgG standards were applied to the three standard curve columns in place of sera. The ELISA plate was then incubated for 2 h at room temperature and then washed 4 times with PBS-T (PBS, 0.05% Tween 20). Alkaline phosphatase-conjugated goat anti-human IgG at 1:1000 dilution was then added to each well and incubated for 1 h. Following this, plates were washed 6 times with PBS-T and 25μl of colorimetric alkaline phosphatase substrate added. Absorbance was measured at 405nm. Antigen-specific IgG concentrations in serum were then calculated based on interpolation from the IgG standard results using a four-parameter logistic (4PL) regression curve fitting model.

### 2.6 Flow cytometry

PBMCs of COVID-19 patients and healthy controls were stained with panels of antibody cocktails as described in **Supplementary Table 2**. Typically, 1–2 x 10^6^ cells were stained per sample. Samples were acquired on a BD LSRFortessa (BD Biosciences) or a Cytek Aurora (Cytek Biosciences).

For cytokine staining, cells were first restimulated. COVID-19 patient samples were incubated in C10 with 10 ng/ml phorbol 12-myristate 13-acetate (PMA, Sigma) and 1 µM ionomycin (Sigma) for four hours at 37°C with 10 µg/ml Brefeldin A (Sigma) added for the last two hours. For cytokine staining of TIGIT^+^ and TIGIT^-^ Tfh after overnight αCD3 stimulation, sorted cells were stimulated overnight at 37°C in C10 in 1 µg/ml αCD3 (clone: OKT3, BioXCell) coated wells with 5 µg/ml αCD28 (clone: CD28.2, Thermo Fisher). 10 ng/ml PMA and 1 µM ionomycin were added for the last four hours and 10 µg/ml Brefeldin A and 2 µM Monensin (Biolegend) for the last two hours. For cytokine staining after coculture assays (as described below), either two or six days after start of the assay 10 ng/ml PMA and 1 µM ionomycin were added for the last four hours and 10 µg/ml Brefeldin A and 2 µM Monensin for the last two hours.

For surface CD40L staining, PBMCs were incubated overnight at 37°C in C10 in the presence of 0.5 µg/ml Staphylococcal Enterotoxin B (SEB). Additionally, 5 µg/ml αCD40 (clone: HB14, Biolegend) or a mouse IgG1κ isotype (clone: P3.6.2.8.1, Thermo Fisher) was added. For intracellular CD40L staining, PBMCs were incubated overnight at 37°C in C10 in the presence of 0.5 µg/ml SEB. For the last two hours of the incubation 10 µg/ml Brefeldin A were added.

### 2.7 Coculture assays

Coculture assays of B cells and Tfh cells were performed as previously described (38). Briefly, B cells and T cells were sorted using a BD FACSAria Fusion (BD Biosciences) (see **Supplementary Table 2** for sorting panels) and cocultured at a 1:1 ratio for six days at 37°C in C10 in the presence of 100 ng/ml SEB. For TIGIT blocking experiments, 1 µg/ml αTIGIT (clone: A15153G, Biolegend) or a mouse IgG2aκ isotype (clone: MOPC-173, Biolegend) was added at the start of the culture. Assays were performed in duplicate.

### 2.8 Data analysis

Flow cytometry data were analysed using FlowJo v.10 (BD Biosciences). For unsupervised clustering, pregated live, singlet lymphocytes were preprocessed in R v.4.0.2 as previously described (5) and the FlowSOM algorithm as implemented in the Bioconductor package CATALYST v.1.14.1 was used to identify CD4 and CD8 T cell populations. FlowSOM clustering was then applied again on these T cell populations and optimal number of clusters was identified using delta area plots. UMAP plots of flow cytometry data were generated from downsampled data using the CATALYST package. Heatmaps were generated using the CATALYST package. Publicly available single cell multi-omics data from the COMBAT study (39) was analysed using the CRAN packages Seurat v.5 (40) and scGate v.1.6 (41). Plots were produced using the CRAN packages ggplot2 v.3.4.4, ggpubr v.0.6.0, ggforestplot v.0.1.0, ggthemes v.5.0.0, gtable v.0.4.3, scales v.1.3.0, colorspace v.2.1–0, cowplot v.1.1.3, scico v.1.5.0, and circlize v.0.4.15. Data cleaning and formatting was carried out using CRAN packages dplyr v.1.1.4, tidyr v.1.3.1, reshape2 v.1.4.4, and Rmisc v.1.5.1. Statistical calculations were performed in R v.4.0.2. Statistical test used and N numbers (number of individuals) are indicated in figure legends. A p value of less than 0.05 was considered significant.

## 3 Results

### 3.1 Severe COVID-19 is associated with distinct T cell phenotypes

**Figure 1:**
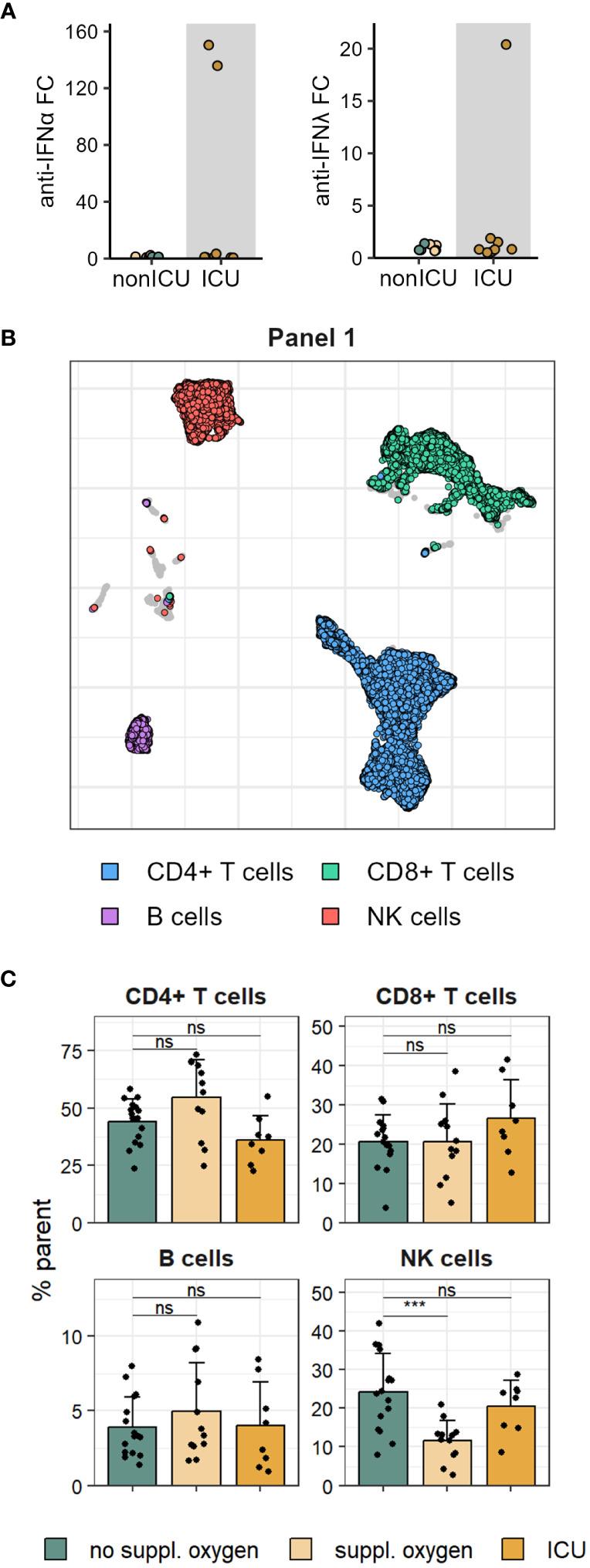
Proportions of major immune cell lineage are mostly unchanged in mild and severe COVID-19. **(A)** Serum anti-IFNα (left) and anti-IFNλ (right) autoantibodies measured in patients admitted to ICU and non-ICU patients. **(B)** UMAP of immune cell subsets in PBMCs stained with flow cytometry Panel 1. **(C)** Frequencies of Panel 1 immune cell subsets shown in B in disease severity groups. Shown are means + SD. No suppl. oxygen, n = 16; Suppl. oxygen, n = 12; ICU, n = 8. Kruskal-Wallis (CD4^+^ T cells, p = 0.022; CD8^+^ T cells, p = 0.370; B cells, p = 0.685; NK cells, p = 0.001) followed by two-tailed Mann–Whitney U-test; ***p < 0.001; ns, not significant.

Cryopreserved PBMCs isolated from the blood of 36 individuals hospitalised with COVID-19 between March and June 2020 were used for deep immunophenotyping using two spectral flow cytometry panels, with matched serum samples analysed for antibody levels. Disease severity was assessed based on supplemental oxygen requirements, with patients exhibiting most severe disease requiring ICU admission. Analysis of anti-IFN autoantibodies revealed two patients with high titres of anti-IFNα autoantibodies, one of which also exhibited anti-IFNλ autoantibodies (**Figure 1A**). Both of these individuals were admitted to ICU consistent with a more severe disease course observed in patients harbouring anti-IFN autoantibodies (27, 28).

**Figure 2:**
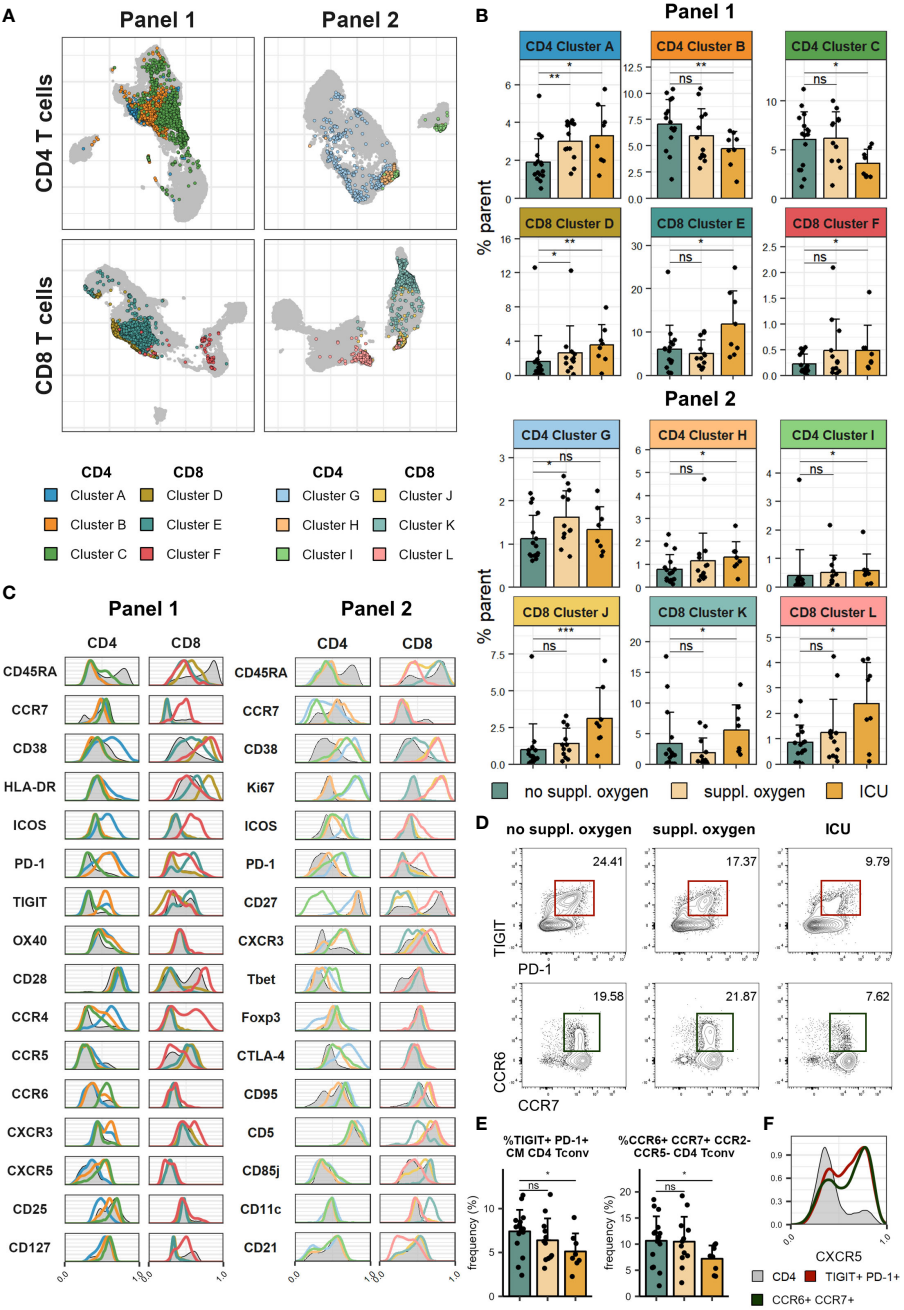
Severe COVID-19 is associated with increased T cell activation and proliferation. Cryopreserved PBMCs of individuals hospitalised with COVID-19 were analysed using two spectral flow cytometry panels. **(A)** UMAP of CD4 (top) and CD8 (bottom) T cells in Panel 1 (left) and Panel 2 (right). Highlighted clusters are significantly different in frequency in disease severity groups. **(B)** Frequencies of clusters within CD4 and CD8 T cells of Panel 1 (top) and Panel 2 (bottom) significantly different between disease severity groups. **(C)** Histograms of marker expression of CD4 and CD8 T cell clusters (colour) and all CD4 and CD8 T cells (grey) in Panel 1 (left) and Panel 2 (right). **(D)** Representative flow cytometry plots of TIGIT^+^PD-1^+^ CM CD4 Tconv (top) and CCR6^+^CCR7^+^CCR2^-^CCR5^-^ CD4 Tconv (bottom). **(E)** Frequency of TIGIT^+^PD-1^+^ CM CD4 Tconv (left) and CCR6^+^CCR7^+^CCR2^-^CCR5^-^ CD4 Tconv (right) in disease severity groups. **(F)** Representative histograms of CXCR5 expression of TIGIT^+^PD-1^+^ CM CD4 Tconv (red), CCR6^+^CCR7^+^CCR2^-^CCR5^-^ CD4 Tconv (green) and all CD4 T cells (grey). In **(B, E)** means + SD are shown. No suppl. oxygen, n = 16; Suppl. oxygen, n = 12; ICU, n = 8. Kruskal-Wallis (Cluster A, p = 0.008; Cluster B, p = 0.051; Cluster C, p = 0.060; Cluster D, p = 0.010; Cluster E, p = 0.050; Cluster F, p = 0.131; Cluster G, p = 0.069; Cluster H, p = 0.110; Cluster I, p = 0.080; Cluster J, p = 0.003; Cluster K, p = 0.034; Cluster L, p = 0.116; TIGIT^+^PD-1^+^ CM CD4 Tconv, p = 0.080; CCR6^+^CCR7^+^CCR2^-^CCR5^-^ CD4 Tconv, p = 0.084) followed by two-tailed Mann–Whitney U-test; ***p < 0.001; **p < 0.01; *p < 0.05; ns, not significant.

Immune cell lineage subsets were identified using FlowSOM clustering (**Figure 1B**, **Supplementary Figures 1A, B**) and no major changes were observed other than a drop in NK cell frequency in patients that received supplemental oxygen (**Figure 1C**, **Supplementary Figures 1C, D**). To better understand the impact of severe COVID-19 on the T cell compartment we performed FlowSOM clustering on CD4 and CD8 T cells from both spectral flow cytometry panels (**Supplementary Figure 2**). Twelve FlowSOM clusters were found to be significantly different in patients that required supplemental oxygen or ICU admission compared to patients with no supplemental oxygen requirements (**Figures 2A–C**). Clusters with elevated frequencies in severe COVID-19 showed high expression of the proliferation marker Ki67 (Panel 2: CD4 clusters G & I and CD8 clusters J & L) as well as activation markers, such as CD38, HLA-DR and ICOS in both CD4 and CD8 T cells (Panel 1: CD4 cluster A and CD8 clusters D, E & F; Panel 2: CD4 clusters G, H & I and CD8 clusters J & L). In some of these clusters we also detected high levels of Tbet and CXCR3 (Panel 2: CD4 cluster I and CD8 clusters J & L), indicative of a type 1 immune response, and lack of CD28 and CD27 expression (Panel 1: CD8 clusters D & E; Panel 2: CD4 cluster I and CD8 clusters J & K), suggestive of a terminally differentiated T cell phenotype. We also identified two clusters of CD4 T cells that were significantly decreased in patients that required ICU admission (Panel 1: CD4 clusters B & C). Further examination of the phenotype of these clusters allowed us to devise manual gating strategies that reflected the frequencies found by FlowSOM clustering (**Figures 2D, E**, **Supplementary Figures 3A, B**). One of these clusters (Panel 1 CD4 cluster B) comprised central memory T cells with high expression of the coinhibitory receptors PD-1 and TIGIT, while the other (Panel 1 CD4 cluster C) was marked by distinctive expression of chemokine receptors, including high expression of CCR6 and CCR7 and lack of CCR2 and CCR5 expression. Of potential interest, both of these clusters were enriched for CXCR5 expression, suggesting that they comprise subsets of cTfh (**Figure 2F**).

**Figure 3:**
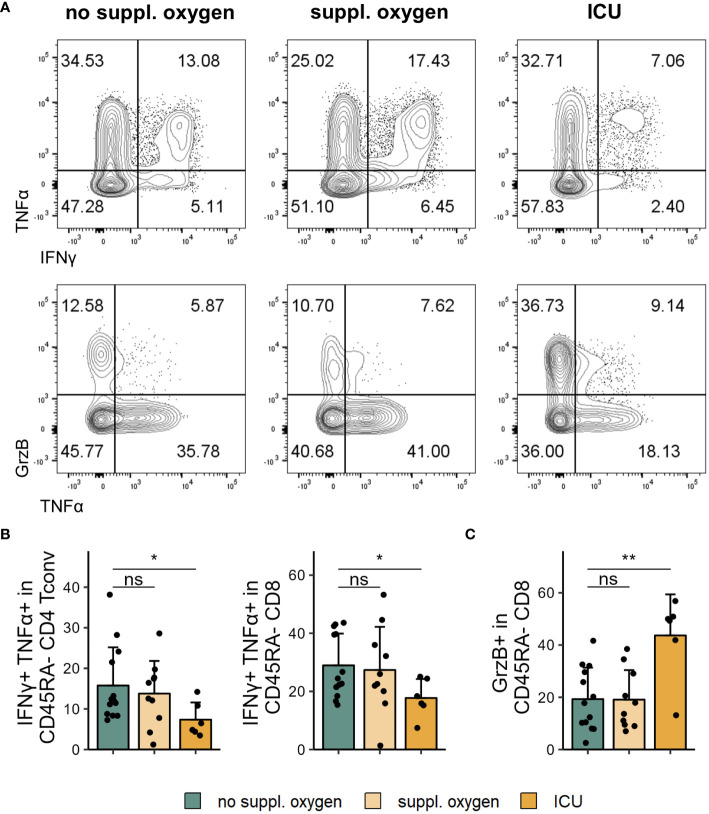
Cytokine profile changes in severe COVID-19. Cryopreserved PBMCs of individuals hospitalised with COVID-19 were restimulated and analysed for cytokine expression using flow cytometry. **(A)** Representative flow cytometry plots of IFNγ^+^TNFα^+^ (top) and GrzB^+^ and TNFα^+^ (bottom) in CD45RA^-^ CD8 T cells. **(B)** Frequencies of IFNγ^+^TNFα^+^CD45RA^-^ CD4 Tconv (left) and CD8 (right) in disease severity groups. **(C)** Frequencies of GrzB^+^CD45RA^-^ CD8 in disease severity groups. Shown are means + SD. No suppl. oxygen, n = 16; Suppl. oxygen, n = 12; ICU, n = 8. Kruskal-Wallis test (IFNγ^+^TNFα^+^CD45RA^-^ CD4 Tconv, p = 0.086; IFNγ^+^TNFα^+^CD45RA^-^ CD8, p = 0.120; GrzB^+^CD45RA^-^ CD8, p = 0.011) followed by two-tailed Mann–Whitney U-test; **p < 0.01; *p < 0.05; ns, not significant.

We examined cytokine expression of T cells following restimulation and found lower frequencies of both CD4 and CD8 T cells that co-expressed IFNγ and TNFα in ICU patients (**Figures 3A, B**). Conversely, the CD8 T cells of these patients exhibited significantly higher levels of granzyme B (**Figures 3A, C**). Granzyme B expression of CD8 T cells correlated positively with frequency of one CD8 T cell cluster identified by FlowSOM (Panel 1 CD8 cluster E, **Supplementary Figure 3C**) which lacked CD28 expression and showed high expression of PD-1 and TIGIT. This cluster could conceivably represent the source of granzyme B expressing CD8 T cells in our dataset.

### 3.2 Higher expression of TIGIT on cTfh is inversely correlated with S1 IgG titres

**Figure 4:**
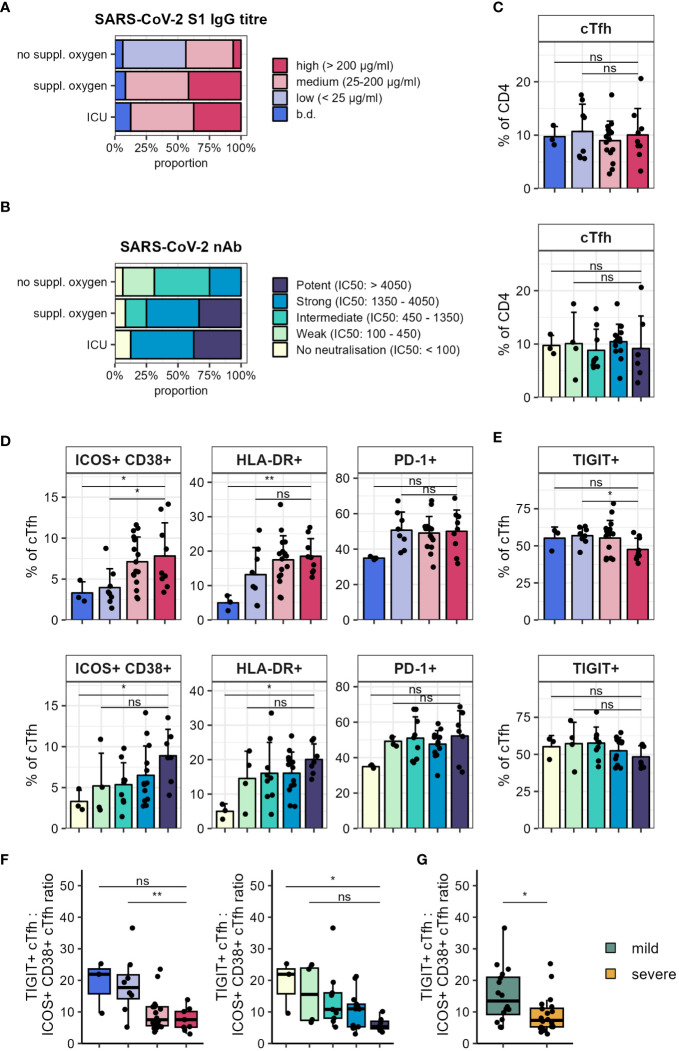
Circulating Tfh subsets correlate with serum anti-SARS-CoV-2 antibodies. Serum S1 IgG titres and neutralising reciprocal ID50 were measured in patients hospitalised with COVID-19. **(A)** Anti-SARS-CoV-2 S1 IgG titre in disease severity groups. **(B)** Anti-SARS-CoV-2 neutralising antibody strength in disease severity groups. **(C)** Frequency of cTfh (CD45RA^-^CXCR5^+^) in S1 IgG titre (top) and nAb strength (bottom) groups. **(D)** Frequency of indicated marker expression in cTfh in S1 IgG titre (top) and nAb strength (bottom) groups. **(E)** Frequency of TIGIT expression in cTfh in S1 IgG titre (top) and nAb strength (bottom) groups. **(F)** Ratio of TIGIT^+^ cTfh to ICOS^+^CD38^+^ cTfh in S1 IgG titre (left) and nAb strength (right) groups. **(G)** Ratio of TIGIT^+^ cTfh to ICOS^+^CD38^+^ cTfh in disease severity groups (mild: no suppl. oxygen; severe: suppl. oxygen + ICU). In **(A, B)** proportions per group are shown. In **(C–E)** means + SD are shown. In **(F, G)** box plots are shown, with black horizontal line denoting median value, while box represents the IQRs (IQR, Q1–Q3 percentile) and whiskers show the minimum (Q1 − 1.5× IQR) and maximum (Q3 + 1.5× IQR) values. High, n = 9; medium, n = 16; low, n = 8; below detection (b.d.), n = 3. Potent, n = 7; strong, n = 13; intermediate, n = 9; weak, n = 4; no neutralisation, n = 3; mild, n = 16; severe, n = 20. Kruskal-Wallis test (S1 IgG cTfh, p = 0.947; nAb cTfh, p = 0.576; S1 IgG ICOS^+^CD38^+^ cTfh, p = 0.018; S1 IgG HLA-DR^+^ cTfh, p = 0.031; S1 IgG PD-1^+^ cTfh, p = 0.127; nAb ICOS^+^CD38^+^ cTfh, p = 0.090; nAb HLA-DR^+^ cTfh, p = 0.066; nAb PD-1^+^ cTfh, p = 0.156; S1 IgG TIGIT^+^ cTfh, p = 0.189; nAb TIGIT^+^ cTfh, p = 0.318; S1 IgG TIGIT^+^ cTfh to ICOS^+^CD38^+^ cTfh, p = 0.015; nAb TIGIT^+^ cTfh to ICOS^+^CD38^+^ cTfh, p = 0.051) followed by two-tailed Mann–Whitney U-test; **p < 0.01; *p < 0.05; ns, not significant.

The generation of robust antibody responses, particularly against the receptor binding domain of the S1 subunit of the spike protein, is important for protection from severe COVID-19 disease upon reinfection (21, 42). Titres of S1 IgG (high: > 200 µg/ml, medium: 25–200 µg/ml, low: < 25 µg/ml) as well as the strength of neutralising antibodies (measured as reciprocal ID50; potent: > 4050, strong: 1350–4050, intermediate: 450–1350, weak: 100–450, no neutralisation: < 100) were previously assessed for this patient cohort (43). When stratifying these by disease severity we found that more severe disease was associated with higher S1 IgG titres as well as stronger neutralising antibodies (**Figures 4A, B**; p=0.0098 and p=0.0133 for S1 IgG titre and nAb respectively, Fisher’s exact test).

Frequencies of cTfh are increased following infection and vaccination (12–14, 26) and SARS-CoV-2 specific Tfh have been shown to correlate with neutralising antibody responses in convalescent individuals (44). We were interested to understand how cTfh frequency and phenotype related to the titres and neutralising strength of SARS-CoV-2 specific antibodies in our patient cohort.

There were no statistically significant changes in the frequencies of cTfh (CD45RA^-^CXCR5^+^, for gating strategy see **Supplementary Figure 4A**) with increased antibody titres and neutralising strengths (**Figure 4C**). However, we found significantly increased frequencies of ICOS and CD38 expressing cTfh from individuals with high S1 IgG titres and stronger neutralising antibodies (**Figure 4D**). Similarly, higher expression of HLA-DR was linked to S1 IgG titre and neutralising strength (**Figure 4D**). While there was a trend for PD-1 expression to be elevated in individuals with detectable S1 IgG this was not statistically significant (**Figure 4D**). Another receptor highly expressed by Tfh is the coinhibitory receptor TIGIT (45), however its role in Tfh function is not well defined. Interestingly, when we probed expression of TIGIT on cTfh we saw that it was significantly lower in individuals with high S1 IgG titres and there was a trend for reduced TIGIT frequencies in individuals with strong neutralising antibodies (**Figure 4E**). This observation was even more striking when comparing TIGIT^+^ cTfh to ICOS^+^CD38^+^ cTfh; the ratio was lower in the high S1 IgG titre and potent neutralising antibody groups (**Figure 4F**). Consistent with the association between severe disease and higher S1 IgG titre and neutralising capacity, we also found that the ratio of TIGIT^+^ cTfh to ICOS^+^CD38^+^ cTfh was lower in severe disease (**Figure 4G**).

**Figure 5:**
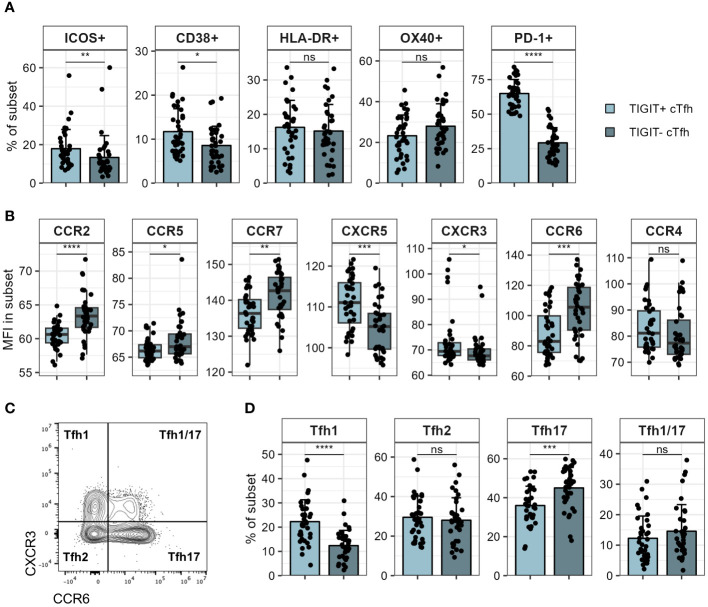
Phenotyping of TIGIT^+^ and TIGIT^-^ cTfh. TIGIT^+^ and TIGIT^-^ cTfh in PBMCs of patients hospitalised with COVID-19 were analysed for expression of various markers using flow cytometry. **(A)** Expression of indicated markers in TIGIT^+^ and TIGIT^-^ cTfh. **(B)** Expression of indicated chemokine receptors in TIGIT^+^ and TIGIT^-^ cTfh. **(C)** Representative flow cytometry plot of CXCR3 and CCR6 in cTfh. Tfh subsets corresponding to chemokine receptor expression pattern are indicated in each quadrant. **(D)** Frequency of indicated Tfh subsets in TIGIT^+^ and TIGIT^-^ cTfh. In **(A, D)** means + SD are shown. In **(B)** box plots are shown, with black horizontal line denoting median value, while box represents the IQRs (IQR, Q1–Q3 percentile) and whiskers show the minimum (Q1 − 1.5× IQR) and maximum (Q3 + 1.5× IQR) values. N = 36. Two-sided Wilcoxon signed-rank test; ****p < 0.0001; ***p < 0.001; **p < 0.01; *p < 0.05; ns, not significant.

Since TIGIT^+^ cTfh were negatively correlated with S1 IgG antibodies, we wondered whether TIGIT expressing and non-expressing cTfh exhibited phenotypic differences that could explain this observation. We therefore examined expression of molecules linked to Tfh function on both TIGIT^+^ and TIGIT^-^ cTfh cells. Against our expectations, we found that TIGIT^+^ cTfh showed slightly higher expression of ICOS and CD38 compared to TIGIT^-^ cTfh while there was no difference in HLA-DR and OX40 levels (**Figure 5A**). TIGIT^+^ cTfh expressed higher levels of PD-1 than TIGIT^-^ cTfh (**Figure 5A**), however, this cannot by itself explain the negative relationship between cTfh TIGIT expression and S1 IgG titres since PD-1 expression on cTfh was not different between individuals with low and high S1 IgG tires, as shown in **Figure 4D**. We went on to examine the chemokine receptor expression of TIGIT^+^ and TIGIT^-^ cTfh and found that TIGIT^+^ cTfh showed higher levels of CXCR5 and CXCR3, while TIGIT^-^ cTfh were higher in expression of CCR7, CCR6, CCR2 and CCR5 (**Figure 5B**). Tfh subsets are classically defined by their expression of the chemokine receptors CXCR3 and CCR6 (46) (**Figure 5C**). We found that TIGIT expressing cTfh were skewed towards a CXCR3^+^CCR6^-^ Tfh1 phenotype while CXCR3^-^CCR6^+^ Tfh17 cells were more prominent in TIGIT^-^ cTfh (**Figure 5D**).

TIGIT expression has been reported on regulatory T cells, including follicular regulatory T cells, which are able to negatively regulate GC responses (45, 47, 48). While we found a significantly higher frequency of CD25^hi^CD127^lo^ cells within TIGIT^+^ compared to TIGIT^-^ cTfh (**Supplementary Figures 4B, C**), excluding these cells from our cTfh gating did not alter the negative association of TIGIT^+^ cTfh with S1 IgG titres (**Supplementary Figure 4D**). Follicular regulatory T cell frequencies and TIGIT expression by follicular regulatory T cells did not differ between groups.

### 3.3 TIGIT expression demarks Tfh with reduced capacity for B cell help and lower production of IL-17 and CD40L

**Figure 6:**
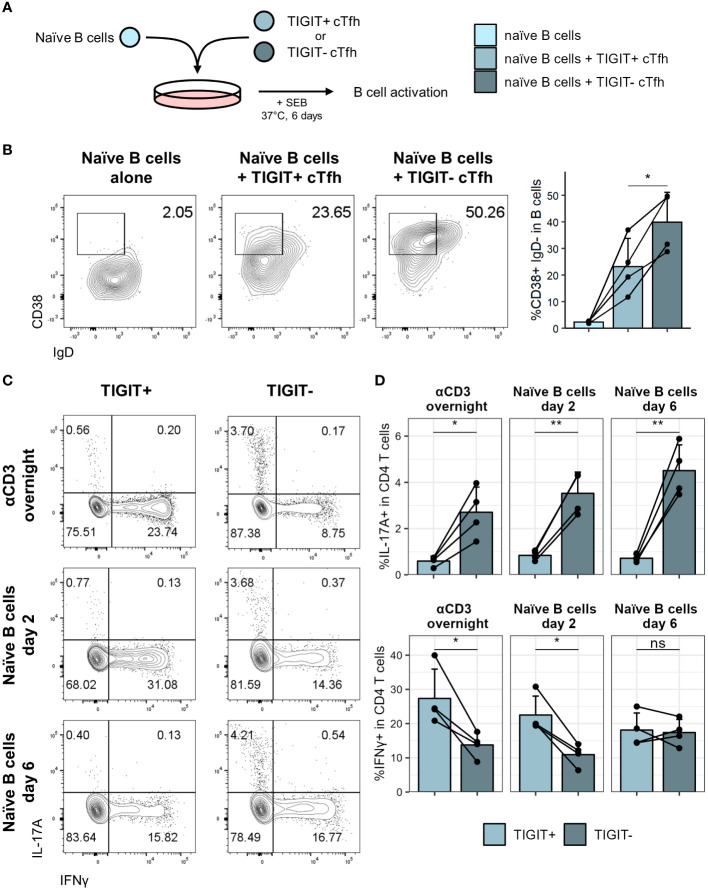
TIGIT^+^ cTfh are poor B cell helpers *in vitro*. TIGIT^+^ and TIGIT^-^ cTfh (CXCR5^+^CD45RA^-^CD4^+^CD3^+^) from PBMCs of self-declared healthy individuals were cocultured with autologous naïve B cells (IgD^+^CD27^-^CD19^+^) to assess B cell helper function. **(A)** T:B coculture setup. **(B)** Representative flow cytometry plots (left) and collated data (right) of CD38^+^IgD^-^ B cells following coculture of naïve B cells with TIGIT^+^ or TIGIT^-^ cTfh. **(C)** Representative flow cytometry plots of IL-17 and IFNγ expression in TIGIT^+^ (left) and TIGIT^-^ (right) cTfh after overnight stimulation (top) or coculture with naïve B cells for two (middle) or six (bottom) days. **(D)** Frequency of IL-17 (top) and IFNγ (bottom) expression in TIGIT^+^ and TIGIT^-^ Tfh following overnight stimulation (left) or coculture with naïve B cells for two (middle) or six (right) days. Shown are means + SD. Data is representative of four independent experiments. N = 4. Two-sided paired Student’s t test; **p < 0.01; *p < 0.05; ns, not significant.

Having established that TIGIT expression on cTfh is lower in individuals with high S1 IgG titres we wondered whether TIGIT^+^ cTfh were perhaps less capable of supporting B cell help than their TIGIT^-^ counterparts. To address this question, we isolated TIGIT^+^ and TIGIT^-^ cTfh as well as naïve B cells from healthy individuals and cocultured them for six days in the presence of staphylococcal enterotoxin B (SEB) (**Figure 6A**). Naïve B cells cultured with TIGIT^-^ cTfh showed greater CD38 upregulation than naïve B cells cultured with TIGIT^+^ cTfh consistent with an increased capacity of TIGIT^-^ cTfh to provide B cell help in this *in vitro* setting (**Figure 6B**). To test whether TIGIT signalling directly impaired B cell helper function of cTfh, we cocultured cTfh and naïve B cells in the presence of a TIGIT blocking antibody. The capacity of cTfh to provide help to B cells was not significantly altered by TIGIT blockade, suggesting that TIGIT expression may instead mark a population of Tfh with reduced helper capacity (**Supplementary Figure 5A**).

To test for phenotypic differences between TIGIT^+^ and TIGIT^-^ cTfh, we assessed expression of a panel of cytokines either after overnight activation with αCD3 or after coculture with naïve B cells for two or six days. It has previously been reported that TIGIT^+^ cTfh produce high levels of IL-21 (49, 50), which can support plasma cell differentiation *in vitro (* 51, 52). While we found a trend of higher IL-21 expression of TIGIT^+^ cTfh after overnight activation, after coculture with naïve B cells for six days TIGIT^-^ cTfh were producing significantly more IL-21 than TIGIT^+^ cTfh (**Supplementary Figure 5B**). At some of the time points examined, TIGIT^-^ cTfh also produced more IL-2, an important factor for T cell survival and proliferation, as well as IL-10, which can also support B cell differentiation (53) (**Supplementary Figure 5B**). We observed no differences in IL-4 expression, another cytokine produced by Tfh (54) (**Supplementary Figure 5B**). Since analysis of chemokine receptor expression had suggested skewing of TIGIT^+^ and TIGIT^-^ cTfh to a Tfh1 and Tfh17 phenotype respectively, we furthermore assessed expression of IFNγ and IL-17. TIGIT^-^ cTfh produced significantly more IL-17 at all time points analysed while TIGIT^+^ cTfh produced almost no IL-17 (**Figures 6C, D**). In contrast, TIGIT^+^ cTfh produced higher levels of IFNγ following overnight stimulation and after two days of coculture with B cells (**Figures 6C, D**). Production of IL-17 and IFNγ is therefore consistent with Tfh subset skewing of TIGIT^+^ and TIGIT^-^ cTfh which we observed in COVID-19 patients (**Figure 5D**) as well as healthy controls (**Supplementary Figure 5C**).

**Figure 7:**
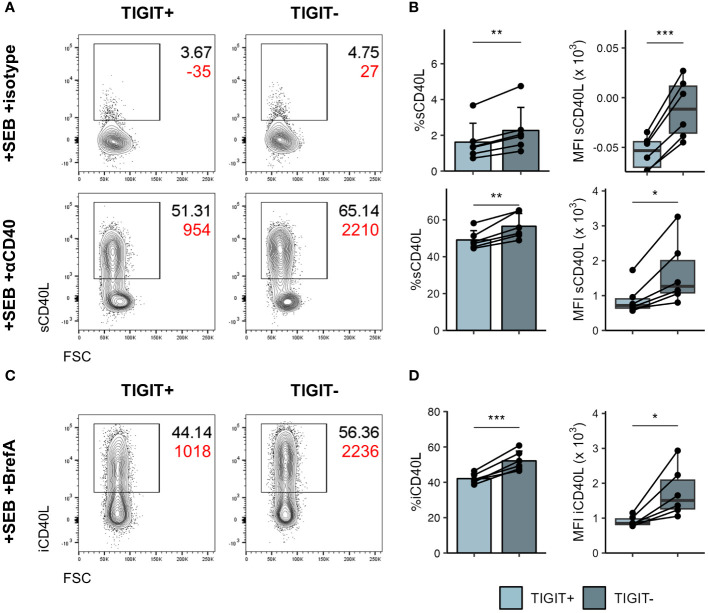
TIGIT^-^ cTfh produce more CD40L following stimulation. PBMCs of self-declared healthy individuals were stimulated overnight with SEB to assess CD40L production. **(A)** Representative flow cytometry plots of surface CD40L expression in TIGIT^+^ (left) and TIGIT^-^ (right) cTfh after overnight stimulation in presence of isotype (top) or anti-CD40 (bottom) antibody. Black text represents frequency, red text represents MFI. **(B)** Collated frequency (left) and MFI (right) of surface CD40L expression in TIGIT^+^ and TIGIT^-^ cTfh in presence of isotype (top) or anti-CD40 (bottom). **(C)** Representative flow cytometry plots of intracellular CD40L expression in TIGIT^+^ (left) and TIGIT^-^ (right) cTfh after overnight stimulation in presence of Brefeldin **(A)** Black text represents frequency, red text represents MFI. **(D)** Frequency (left) and MFI (right) of intracellular CD40L expression in TIGIT^+^ and TIGIT^-^ cTfh. In **(B, D)** on the left means + SD are shown. In **(B, D)** on the right box plots are shown, with black horizontal line denoting median value, while box represents the IQRs (IQR, Q1–Q3 percentile) and whiskers show the minimum (Q1 − 1.5× IQR) and maximum (Q3 + 1.5× IQR) values. Data is representative of two independent experiments. N = 6. Two-sided paired Student’s t test; ***p < 0.001; **p < 0.01; *p < 0.05.

To further understand phenotypic differences between TIGIT^+^ and TIGIT^-^ cTfh, we made use of the publicly available multi-omics dataset of peripheral blood immune cells from COVID-19 patients collected by the COMBAT consortium (39). Tfh that egress from lymphoid tissues and enter the blood downregulate most Tfh associated markers; while CXCR5 expression appears least affected (55), nonetheless, *CXCR5* mRNA expression is sparsely detected in peripheral blood CD4 T cells (**Supplementary Figure 6A**) and it can be difficult to identify cTfh based on transcriptomics alone. Instead, we made use of CITE-seq protein expression data available within the COMBAT dataset and the single cell signature scoring algorithms implemented in scGate (41) to identify CXCR5^+^CD45RA^-^ Tfh-like cells in this dataset (**Supplementary Figures 6B–D**). Using the same approach to identify TIGIT^+^ and TIGIT^-^ cells within the Tfh-like cells (**Supplementary Figures 6E–G**) we then went on to identify genes differentially expressed between these two subsets (**Supplementary Figure 6H**). As expected *TIGIT* was expressed at higher levels within the TIGIT^+^ cells and we could also confirm CXCR3 and CCR6 skewing on the mRNA level. Furthermore, TIGIT^+^ cells expressed higher levels of Tfh associated markers *ICOS*, *PDCD1* and *TOX*. Conversely, TIGIT^-^ cells showed higher expression of the proliferation marker *MYC* and also interestingly the costimulatory molecule *CD40LG*. Following *in vitro* stimulation with SEB we could confirm that both surface as well as intracellular levels of CD40L were significantly higher in TIGIT^-^ cTfh compared to TIGIT^+^ cTfh (**Figure 7**). Given the importance of CD40L/CD40 interactions in driving B cell activation and differentiation in T cell dependent antibody responses, increased expression of CD40L could conceivably contribute to the superior B-helper function in TIGIT^-^ cTfh cells.

Thus, TIGIT demarks cTfh that have reduced capability for activating B cells *in vitro* and produce lower levels of IL-17 and CD40L compared to their TIGIT^-^ counterparts.

## 4 Discussion

The quality of the antibody response is profoundly shaped by the nature of the T cells providing B cell help. Here, we have identified a relationship between subsets of peripheral blood Tfh and the anti-S1 serum antibody response in a cohort of patients hospitalised with COVID-19. While the main role of Tfh is within the GC, counterparts of these GC Tfh can be found in the circulation (6) and links between cTfh subsets, particularly ICOS and CD38 expressing cTfh, and antigen specific antibody responses have previously been reported following vaccination (12, 56, 57). We corroborate these findings here in the setting of SARS-CoV-2 infection, showing higher frequencies of cTfh expressing ICOS, CD38 as well as HLA-DR within the peripheral blood of individuals with higher S1 IgG titres and stronger neutralising antibodies. Furthermore, we find a previously unreported, negative association between the anti-S1 antibody response and cTfh expressing the coinhibitory receptor TIGIT, with higher frequencies of TIGIT expressing cTfh found in individuals with lower S1 IgG titres. Tfh are known to express high levels of TIGIT, particularly within the GC. Its role in Tfh function, however, is incompletely understood although it has been suggested that within the GC it may insulate Tfh from signals received through CD226, a costimulatory receptor that shares its ligands with TIGIT (58).

We further demonstrate that TIGIT^-^ cTfh are superior to TIGIT^+^ cTfh in their ability to activate B cells in *in vitro* coculture assays. This does not appear to be mediated by TIGIT itself as TIGIT blocking antibodies had no effect on Tfh mediated B cell activation. Indeed, Yasutomi et al. showed that CD112 and CD155, the shared ligands of TIGIT and CD226, are not expressed by tonsillar B cells. Instead, these ligands are predominantly expressed by CD14^+^ monocytes and CD11c^+^ conventional dendritic cells (58), making these antigen presenting cells the more likely interaction partner for both CD226 and TIGIT expressed on T cells. TIGIT therefore appears to instead demark a population of cTfh cells that are less capable of providing help during B cell activation. We show that following *in vitro* activation TIGIT^+^ cTfh express lower levels of CD40L, a critical mediator of T-dependent (TD) antibody responses known to promote B cell activation, proliferation and differentiation (59). While both TIGIT^+^ and TIGIT^-^ cTfh were able to upregulate CD40L following activation, TIGIT^-^ cTfh did so to a higher extent. This difference in CD40L expression could therefore, at least partially, explain the difference in B cell activation observed in coculture assays.

Aside from CD40L, we also found that TIGIT^+^ and TIGIT^-^ cTfh displayed a distinct cytokine profile. Cytokines produced by Tfh play a key role in shaping TD antibody responses and of these the importance of IL-21 and IL-4 in shaping the GC reaction is well recognised (60, 61). Previous reports suggested that TIGIT expressing cTfh produce high levels of IL-21 following *in vitro* stimulation (49, 50). While we did see a trend for higher amounts of IL-21 following overnight activation, in the longer coculture assays we found TIGIT^-^ cTfh produced more IL-21 than TIGIT^+^ cTfh, with no difference in IL-4 production. Furthermore, we identified that IFNγ and IL-17 were differentially expressed in TIGIT^+^ and TIGIT^-^ cTfh. Both cytokines can be produced by Tfh and have been shown to influence GC reactions, with IL-17 reported to promote TD antibody responses (62–64) while the role of IFNγ appears to be more ambiguous (65–69). These cytokines are also typically linked to Th1 and Th17 subsets and, in line with this, we found IFNγ producing TIGIT^+^ cTfh to be predominantly CXCR3^+^CCR6^-^ Tfh1, while IL-17 producing TIGIT^-^ cTfh were skewed towards a CXCR3^-^CCR6^+^ Tfh17 phenotype. Interestingly, a recent study by He et al. found that CXCR3^-^ cTfh (isolated from healthy, COVID-19 convalescent individuals or vaccine recipients) were superior in their capacity to activate B cells in coculture compared with their CXCR3^+^ cTfh counterparts (70), mirroring our observations in the TIGIT^+^ versus TIGIT^-^ cTfh compartments. Thus, a preference for cytokine usage in particular Tfh subsets may be another contributing factor to the superior ability of TIGIT^-^ cTfh to promote B cell activation *in vitro*.

The demonstration that severe COVID-19 is associated with higher titres of neutralising S1 IgG as well as increased frequencies of CD4 and CD8 T cells expressing CD38, HLA-DR and Ki67 is consistent with reports from other patient cohorts where increased immune activation was linked to severe disease (25, 36, 43, 71). It is difficult to disentangle whether these observations are linked to an overactive, and perhaps inappropriate, immune response or are rather due to higher viral loads that may have been present in individuals experiencing more severe disease, perhaps facilitated by a diminished NK cell population (72). Nonetheless, these findings suggest the possibility of using peripheral immune cell subsets as biomarkers for disease severity.

A major outstanding question remains regarding how peripheral blood TIGIT cTfh subsets relate to Tfh in secondary lymphoid organs. While clonal relationships between circulating and GC Tfh have been established (3, 73), it is thought that cTfh predominantly arise from pre-Tfh at the T-B border and not the GC itself, demonstrated by the presence of cTfh in SAP deficient individuals with impaired T-B interactions (2). Results published by Yasutomi et al. provide insight into transcriptional differences of TIGIT^+^ and TIGIT^-^ Tfh isolated from human tonsils (58). They conclude that TIGIT^+^ Tfh are more differentiated and less proliferative than TIGIT^-^ Tfh and have a GC Tfh phenotype. During an ongoing immune response an influx of activated ICOS^+^CD38^+^ but TIGIT^-^ pre-Tfh could conceivably shift the balance of TIGIT^-^ and TIGIT^+^ cTfh within the circulation, leading to a proportional decrease in TIGIT^+^ cTfh. Future work investigating the clonal relationship between TIGIT^+^ and TIGIT^-^ Tfh in secondary lymphoid organs and the blood would provide further insight here, and inclusion of TIGIT analysis will enhance efforts to exploit cTfh as a biomarker population in the context of vaccination, infection and autoimmunity.

## Data availability statement

The raw data supporting the conclusions of this article will be made available by the authors, without undue reservation.

## Ethics statement

The studies involving humans were approved by UCL–Royal Free Hospital BioBank Ethical Review Committee Reference number: NC2020.24 NRES EC number: 16/WA/0289. The studies were conducted in accordance with the local legislation and institutional requirements. The participants provided their written informed consent to participate in this study.

## Author contributions

NE: Formal Analysis, Investigation, Methodology, Visualization, Writing – original draft, Writing – review & editing. LH: Investigation, Writing – review & editing. EN: Investigation, Methodology, Writing – review & editing. CR-S: Investigation, Writing – review & editing. LP: Investigation, Writing – review & editing. CW: Investigation, Writing – review & editing. AF: Investigation, Writing – review & editing. YE: Investigation, Writing – review & editing. AR: Investigation, Writing – review & editing. RB: Data curation, Writing – review & editing. KK: Investigation, Writing – review & editing. PP: Investigation, Writing – review & editing. LM: Funding acquisition, Supervision, Writing – review & editing. LW: Conceptualization, Funding acquisition, Methodology, Project administration, Supervision, Writing – original draft, Writing – review & editing.
