# Supplementary material for: TIGIT+Tfh show poor B-helper function and negatively correlate with SARS-CoV-2 antibody titre
Source: Front Immunol. 2024 May 29;15:1395684. doi: 10.3389/fimmu.2024.1395684 (PMC11167088; doi:10.3389/fimmu.2024.1395684)
Supplement: Supplementary file 3 [file DataSheet_1.docx]

Supplementary Material

# Supplementary Figures


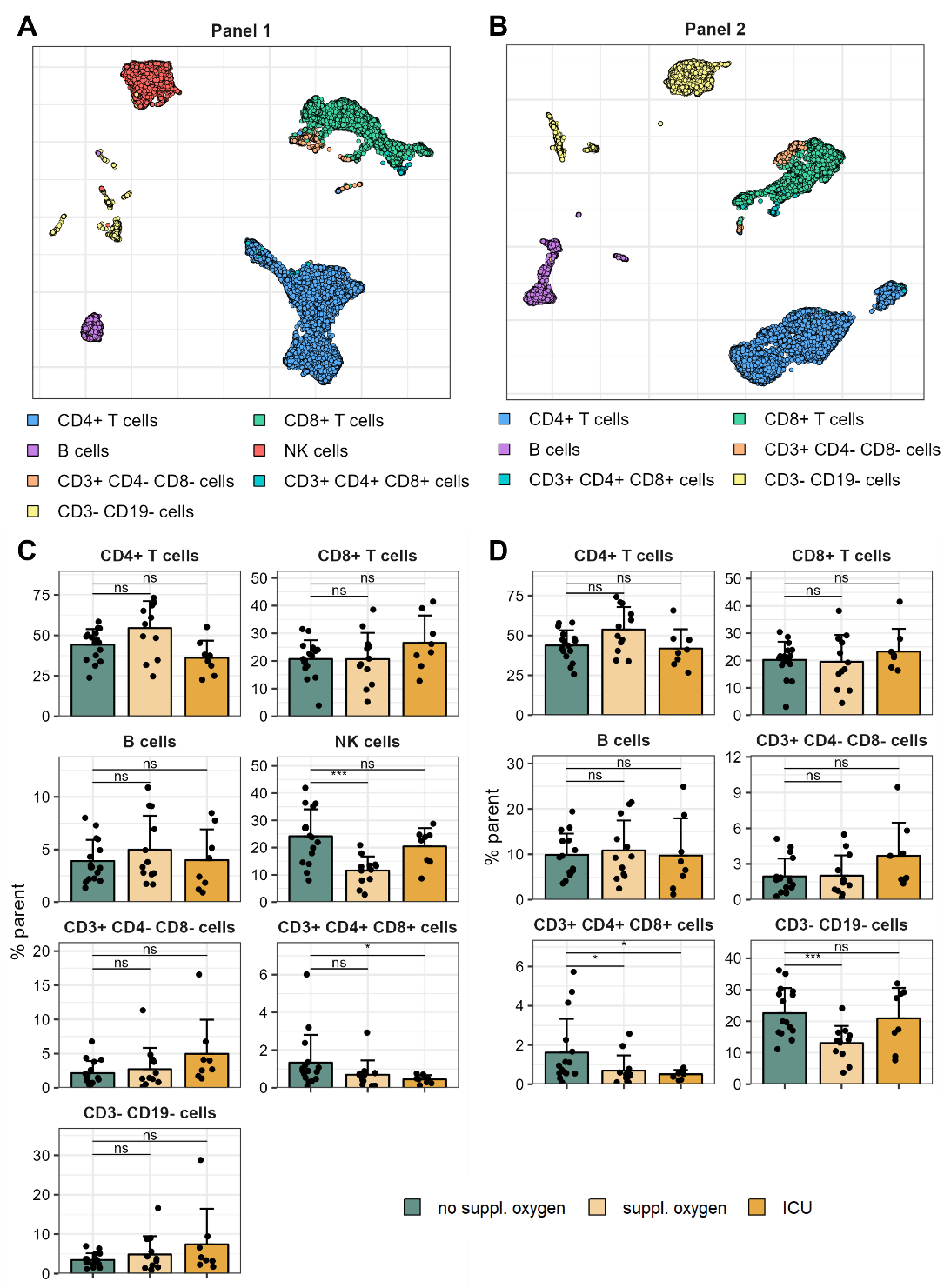


**Supplementary Figure 1.** (A) UMAP of Panel 1 cells showing FlowSOM clusters. (B) UMAP of Panel 2 cells showing FlowSOM clusters. (C) Frequencies of Panel 1 FlowSOM clusters in disease severity groups. (D) Frequencies of Panel 2 FlowSOM clusters in disease severity groups. Shown are means + SD. No suppl. oxygen, n = 16; Suppl. oxygen, n = 12; ICU, n = 8. Kruskal-Wallis test (Panel 1: CD4^+^ T cells, p = 0.022; CD8^+^ T cells, p = 0.370; B cells, p = 0.685; NK cells, p = 0.001; CD3^+^CD4^-^CD8^-^ cells, p = 0.130; CD3^+^CD4^+^CD8^+^ cells, p = 0.035; CD3^-^CD19^-^ cells, p = 0.529; Panel 2: CD4^+^ T cells, p = 0.087; CD8^+^ T cells, p = 0.742; B cells, p = 0.804; CD3^+^CD4^-^CD8^-^ cells, p = 0.141; CD3^+^CD4^+^CD8^+^ cells, p = 0.041; CD3^-^CD19^-^ cells, p = 0.006) followed by two-tailed Mann–Whitney U-test; ***, p < 0.001; *, p < 0.05; ns, not significant.


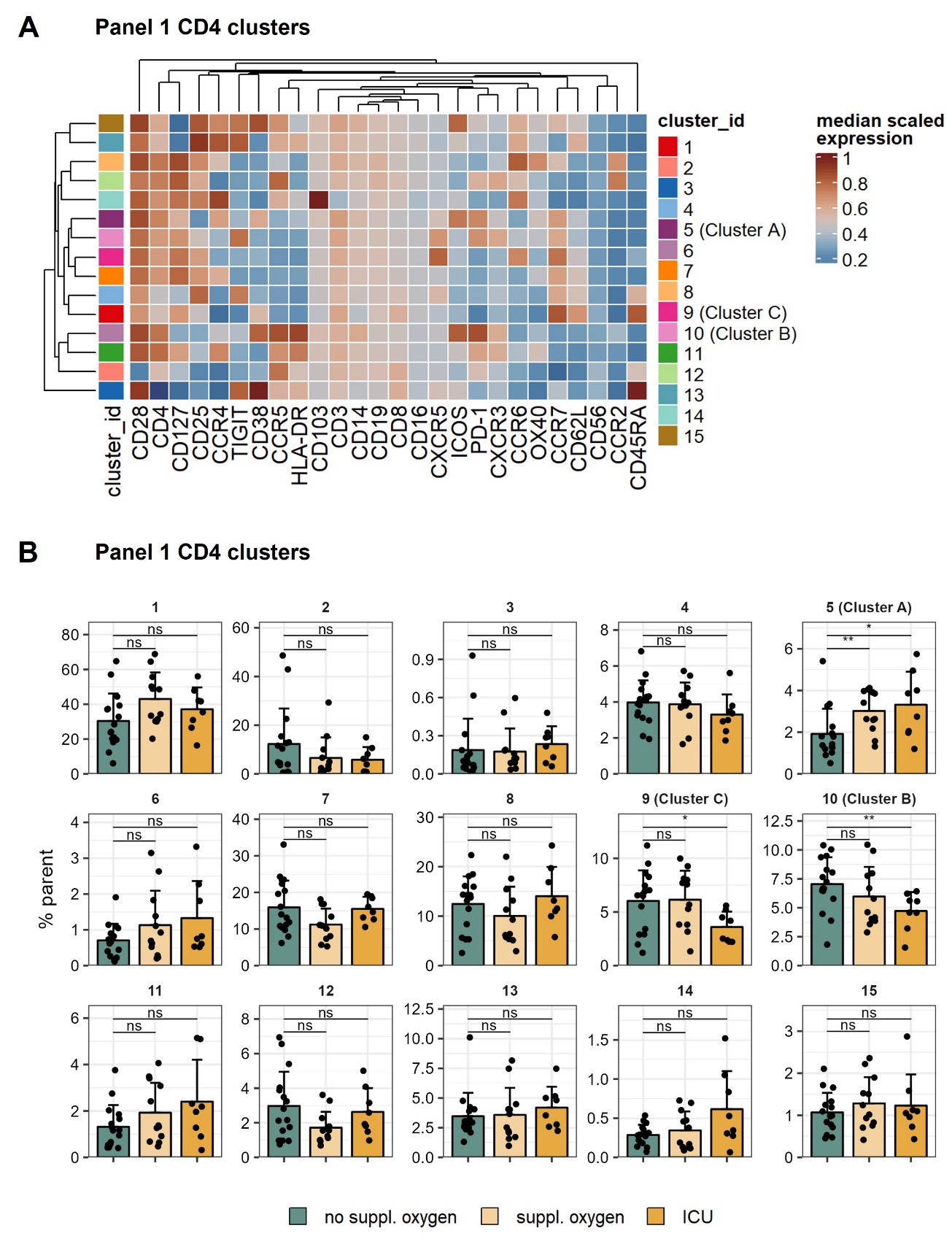


**Supplementary Figure 2.**


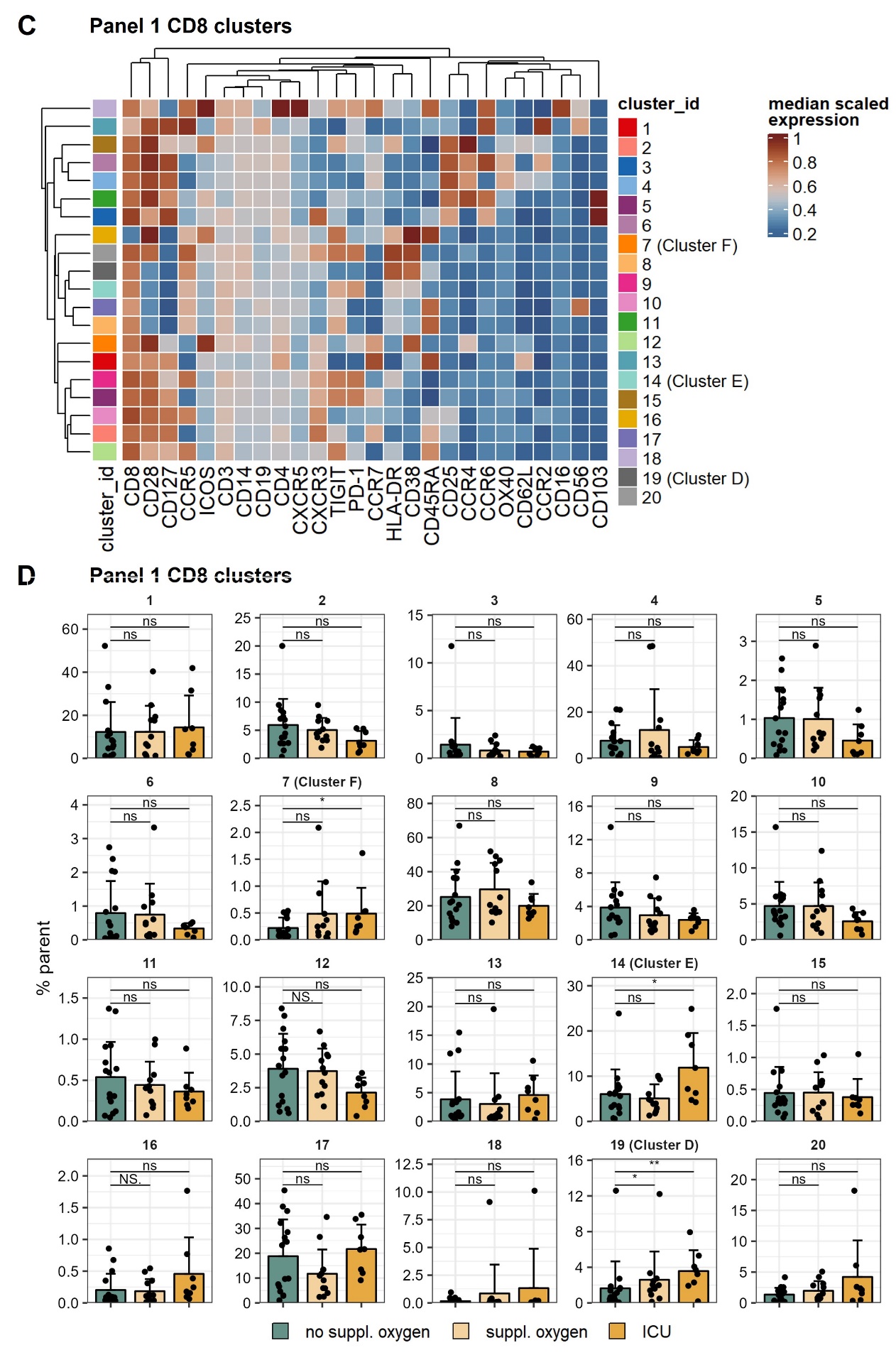


**Supplementary Figure 2 (cont).**


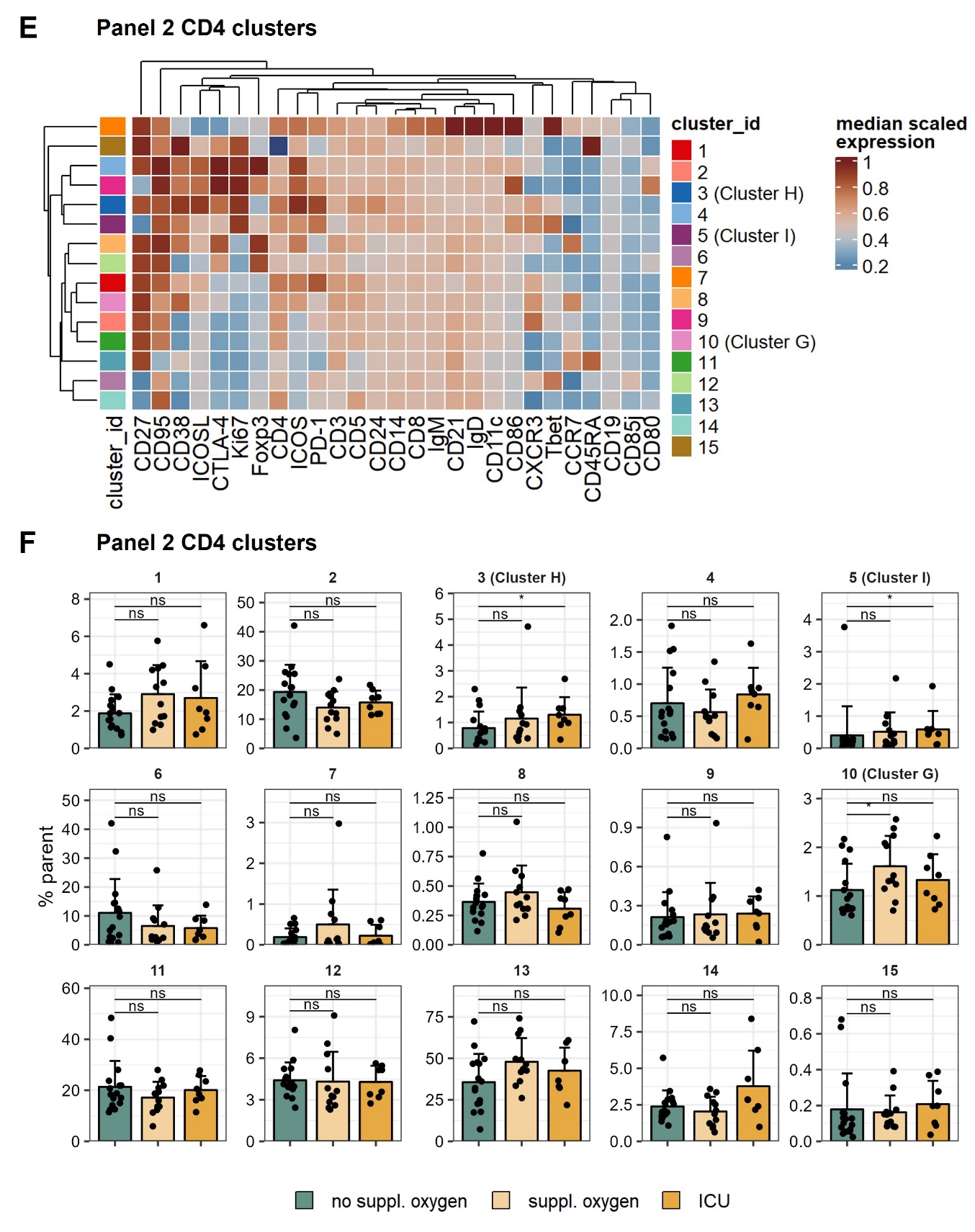


**Supplementary Figure 2 (cont).**


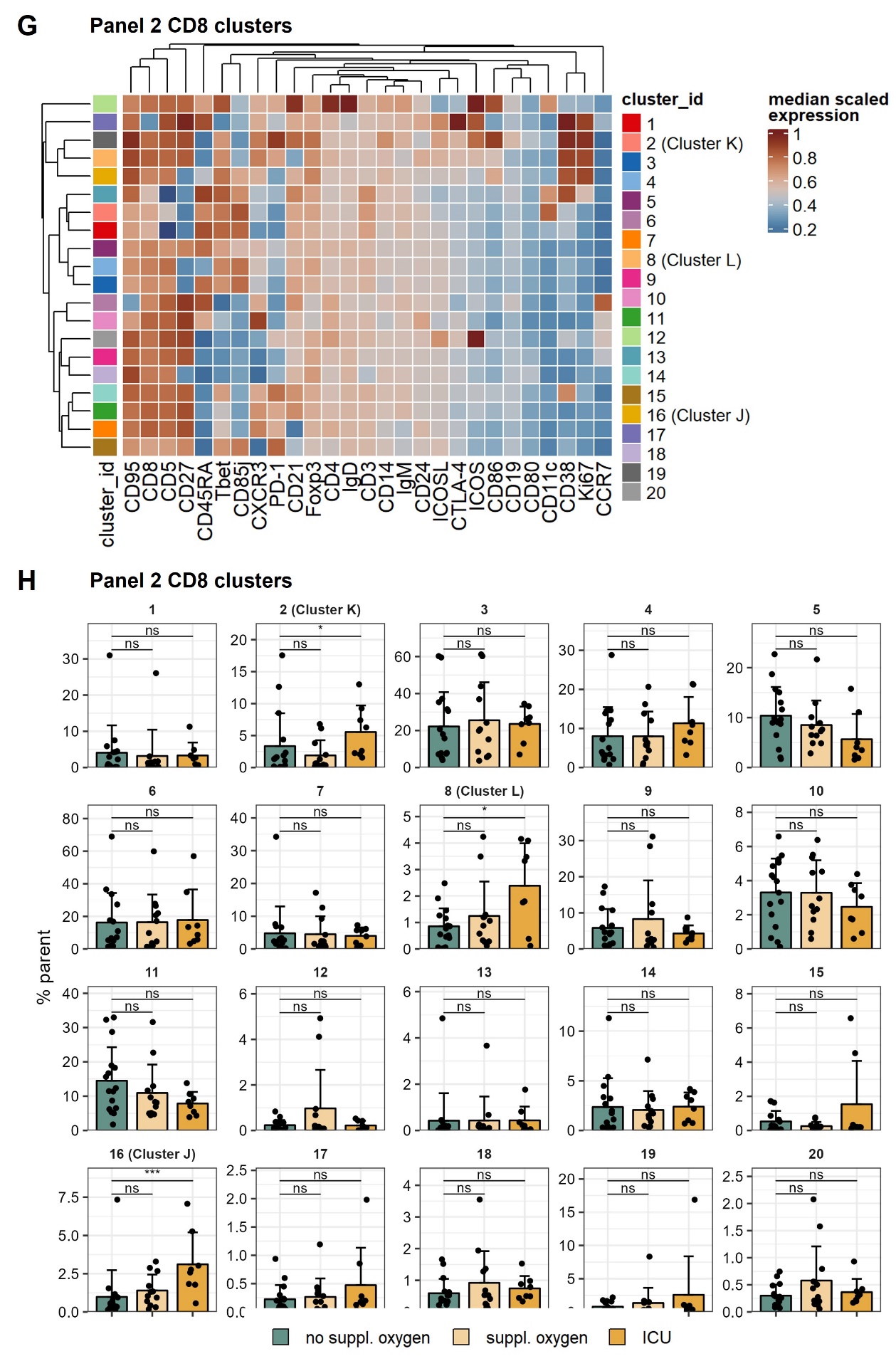


**Supplementary Figure 2 (cont).**

**Supplementary Figure 2.** (A) Heatmap of Panel 1 marker expression in CD4 FlowSOM clusters. (B) Frequencies of Panel 1 CD4 FlowSOM clusters in disease severity groups. (C) Heatmap of Panel 1 marker expression in CD8 FlowSOM clusters. (D) Frequencies of Panel 1 CD8 FlowSOM clusters in disease severity groups. (E) Heatmap of Panel 2 marker expression in CD4 FlowSOM clusters. (F) Frequencies of Panel 2 CD4 FlowSOM clusters in disease severity groups. (G) Heatmap of Panel 2 marker expression in CD8 FlowSOM clusters. (H) Frequencies of Panel 2 CD8 FlowSOM clusters in disease severity groups. Shown are means + SD. No suppl. oxygen, n = 16; Suppl. oxygen, n = 12; ICU, n = 8. Kruskal-Wallis test (Panel 1 CD4 clusters: 1, p = 0.122; 2, p = 0.550; 3, p = 0.313; 4, p = 0.245; 5, p = 0.008; 6, p = 0.378; 7, p = 0.094; 8, p = 0.328; 9, p = 0.060; 10, p = 0.051; 11, p = 0.239; 12, p = 0.193; 13, p = 0.359; 14, p = 0.267; 15, p = 0.816; Panel 1 CD8 clusters: 1, p = 0.873; 2, p = 0.140; 3, p = 0.917; 4, p = 0.830; 5, p = 0.095; 6, p = 0.709; 7, p = 0.131; 8, p = 0.369; 9, p = 0.329; 10, p = 0.131; 11, p = 0.695; 12, p = 0.119; 13, p = 0.236; 14, p = 0.050; 15, p = 0.933; 16, p = 0.202; 17, p = 0.155; 18, p = 0.959; 19, p = 0.010; 20, p = 0.246; Panel 2 CD4 clusters: 1, p = 0.210; 2, p = 0.210; 3, p = 0.107; 4, p = 0.261; 5, p = 0.079; 6, p = 0.520; 7, p = 0.508; 8, p = 0.399; 9, p = 0.589; 10, p = 0.069; 11, p = 0.553; 12, p = 0.593; 13, p = 0.140; 14, p = 0.168; 15, p = 0.356; Panel 2 CD8 clusters: 1, p = 0.464; 2, p = 0.034; 3, p = 0.802; 4, p = 0.366; 5, p = 0.091; 6, p = 0.938; 7, p = 0.811; 8, p = 0.116; 9, p = 0.932; 10, p = 0.544; 11, p = 0.193; 12, p = 0.661; 13, p = 0.487; 14, p = 0.573; 15, p = 0.483; 16, p = 0.003; 17, p = 0.695; 18, p = 0.578; 19, p = 0.975; 20, p = 0.573) followed by two-tailed Mann–Whitney U-test; ***, p < 0.001; **, p < 0.01; *, p < 0.05; ns, not significant.


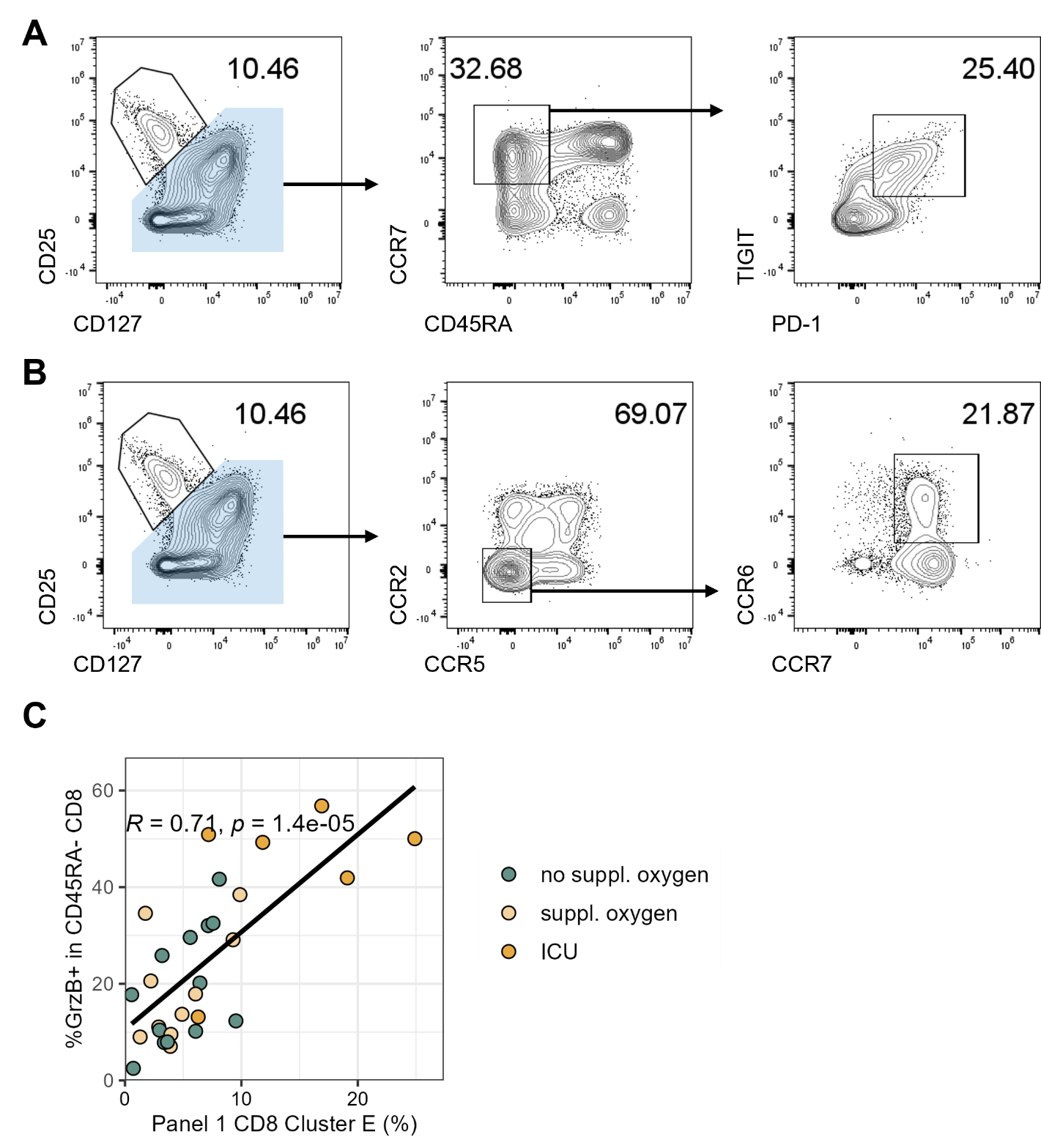


**Supplementary Figure 3.** (A) Representative gating strategy for TIGIT^+^PD-1^+^ CM CD4 Tconv. Light blue box represents Tconv gate (NOT CD127^lo^CD25^hi^ Treg). (B) Representative gating strategy for CCR6^+^CCR7^+^CCR2^-^CCR5^-^ CD4 Tconv. Light blue box represents Tconv gate (NOT CD127^lo^CD25^hi^ Treg). (C) Pearson correlation of GrzB^+^CD45RA^-^ CD8 T cell frequency and Panel 1 CD8 Cluster E frequency. No suppl. oxygen, n = 16; Suppl. oxygen, n = 12; ICU, n = 8. Pearson’s R and associated p value are depicted on plot. Black line only for visualisation purposes.


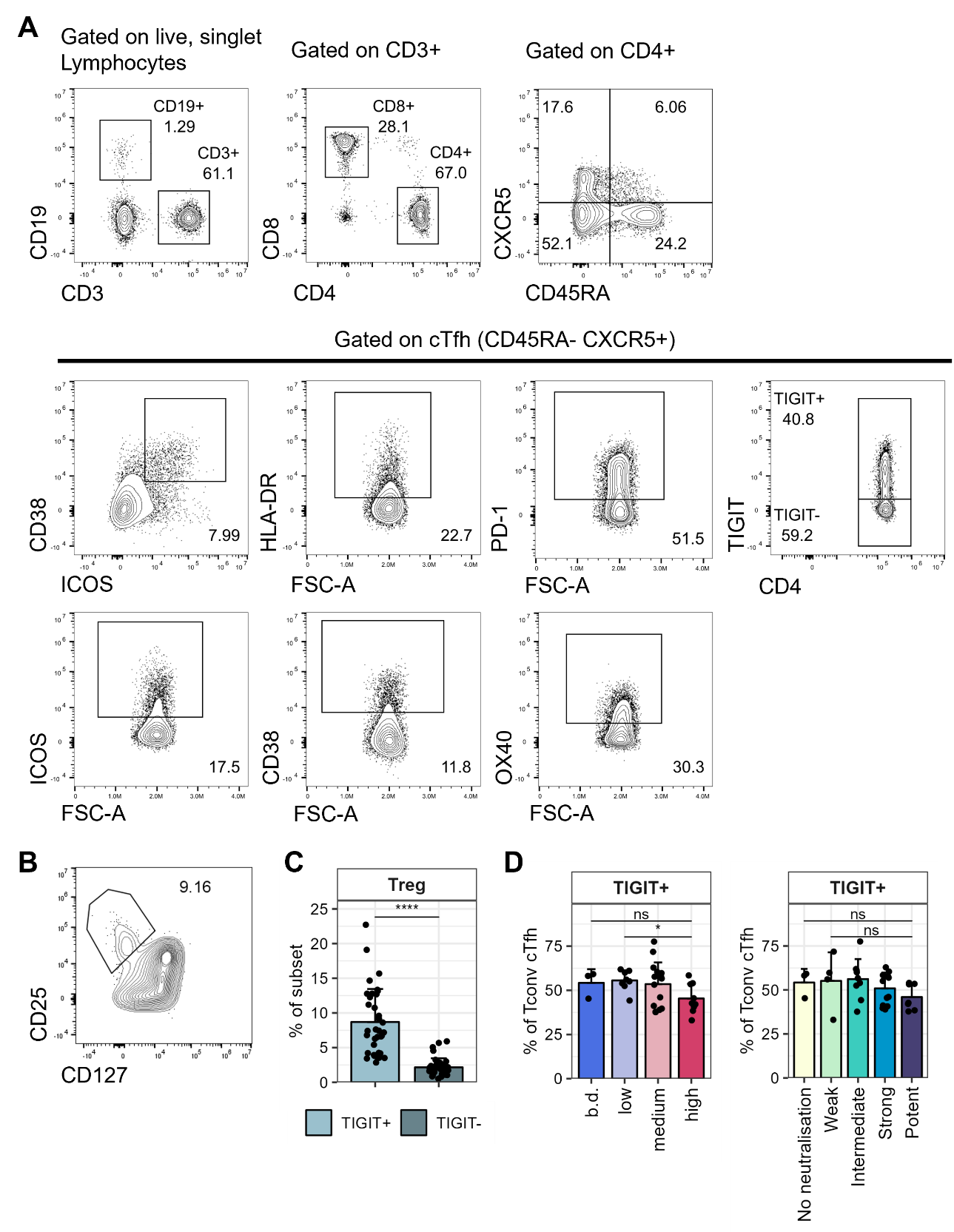


**Supplementary Figure 4.** (A) Representative gating strategy for cTfh (CD45RA^-^CXCR5^+^) and cTfh marker expression. (B) Representative flow cytometry plot of Treg (CD127^lo^CD25^hi^) in cTfh. (C) Frequency of Treg in TIGIT^+^ and TIGIT^-^ cTfh. N = 36. (D) Frequency of TIGIT^+^ Tconv Tfh in S1 IgG titre (left) and nAb strength (right) groups. High, n = 9; medium, n = 16; low, n = 8; below detection (b.d.), n = 3. Potent, n = 7; strong, n = 13; intermediate, n = 9; weak, n = 4; no neutralisation, n = 3. Kruskal-Wallis test (S1 IgG TIGIT^+^ Tconv Tfh, p = 0.158; nAb TIGIT^+^ Tconv Tfh, p = 0.274) followed by two-tailed Mann–Whitney U-test; ****, p < 0.0001; *, p < 0.05; ns, not significant.


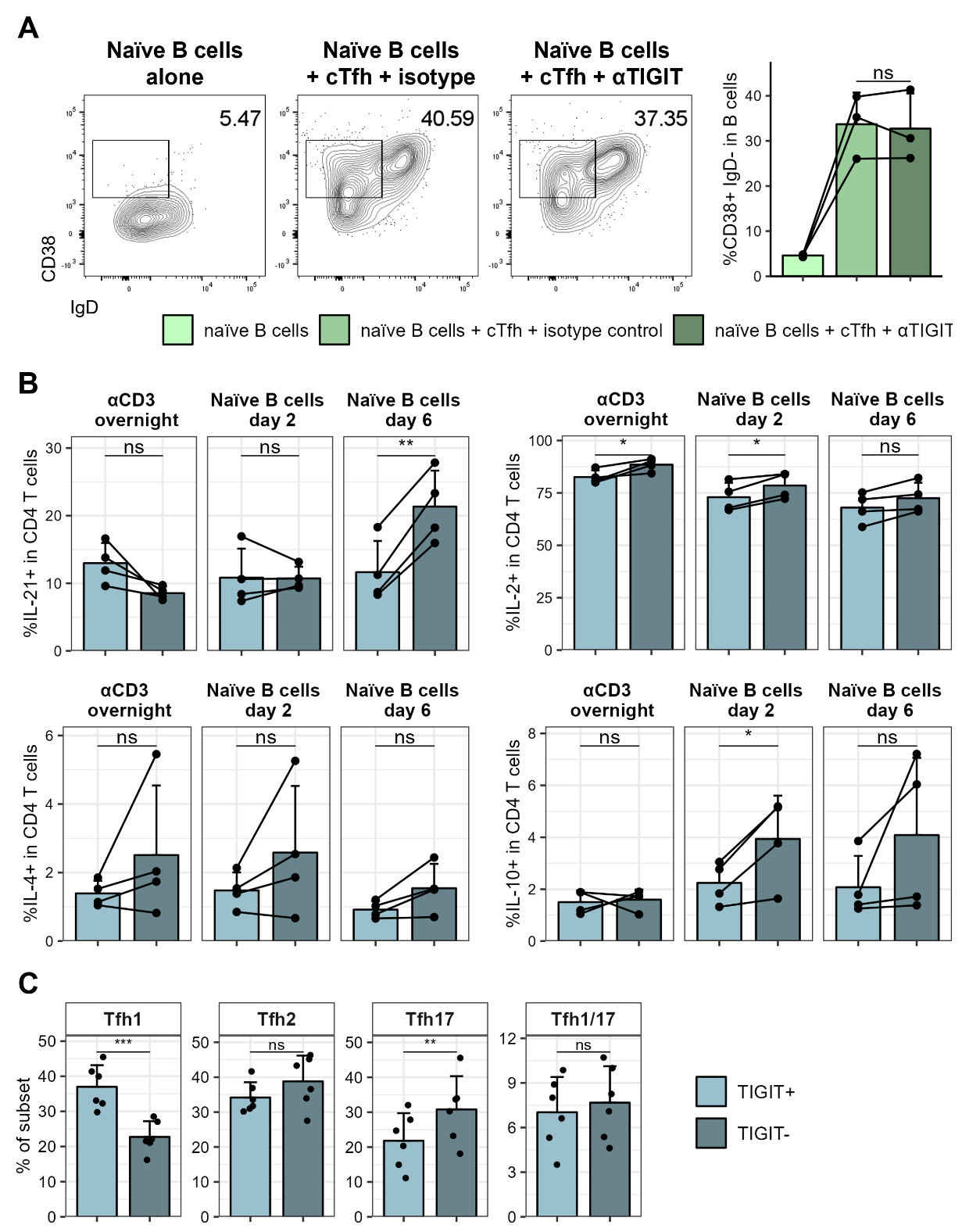


**Supplementary Figure 5.** (A) Representative flow cytometry plots (left) and collated data (right) of CD38^+^IgD^-^ B cells following coculture of naïve B cells with cTfh in presence of isotype (middle) or anti-TIGIT (right) antibody. Data is representative of three independent experiments. N = 3. (B) Frequency of indicated cytokine expression in TIGIT^+^ and TIGIT^-^ cTfh following overnight stimulation (left) or coculture with naïve B cells for two (middle) or six (right) days. Data is representative of four independent experiments. N = 4. (C) Frequency of indicated Tfh subsets in TIGIT^+^ and TIGIT^-^ cTfh in PBMCs of healthy controls. Data is representative of two independent experiments. N = 6. Two-sided paired Student’s t test; ***, p < 0.001; **, p < 0.01; *, p < 0.05; ns, not significant.


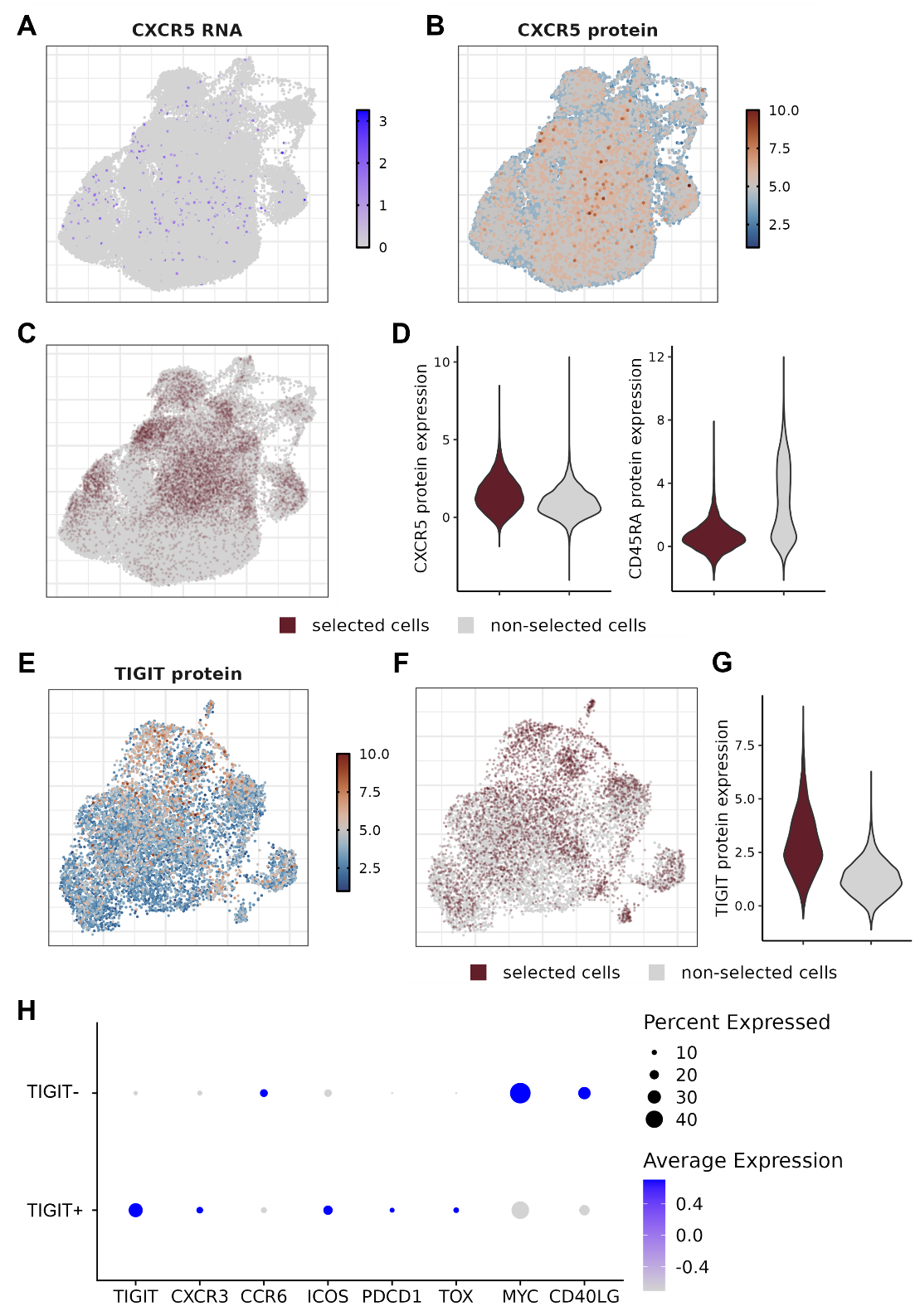


**Supplementary Figure 5.** Single cell multi-omics data of CD4 T cells collected from individuals with COVID-19 by the COMBAT consortium were used to study transcriptomic differences in TIGIT^+^ and TIGIT^‑^ cTfh. (A/B/C) UMAP of CD4 T cells analysed. Colour shows *CXCR5* RNA (A) or CXCR5 protein (B) expression or Tfh-like cells selected by scGate based on CXCR5^+^ and CD45RA^-^ protein expression (16.08%) (C). (D) Violin plots showing CXCR5 (left) and CD45RA (right) protein expression in selected and non-selected cells shown in (C). (E/F) UMAP of Tfh-like cells selected by scGate. Colour shows TIGIT protein expression (E) or cells selected by scGate based on TIGIT protein expression (45.21%) (F). (G) Violin plots showing TIGIT protein expression in selected and non-selected cells shown in (F). (H) Dot plot of subset of differentially expressed genes in TIGIT^+^ and TIGIT^-^ Tfh-like cells.

# Supplementary Table legends

**Suppl. Table 1** Information on patient cohort

**Suppl. Table 2** Flow cytometry antibody panels
